# Clinical trends and utilization of clear aligner therapy among specialist orthodontists in Iran

**DOI:** 10.3389/froh.2026.1858028

**Published:** 2026-06-18

**Authors:** Ali Borzabadi-Farahani

**Affiliations:** Department of Orthodontics, School of Dentistry, Cardiff University, Cardiff, Wales, United Kingdom

**Keywords:** clear aligner therapy, Iran, orthodontic practice, orthodontics, survey

## Abstract

**Objective:**

Clear aligner therapy (CAT) has transformed contemporary orthodontic practice; however, evidence regarding its adoption and clinical use among specialist orthodontists remains scarce. This study investigated CAT practices among Iranian specialist orthodontists.

**Methodology:**

This study forms part of the Cardiff University International Aligner Survey: Orthodontists” Practices and Perceptions. A web-based cross-sectional survey of specialist orthodontists in Iran was conducted from October 2025 to January 2026. A voluntary, anonymous English-language questionnaire assessed demographics, CAT usage patterns, adjuncts, training, preferred malocclusions and patient age groups, monitoring and retention protocols, and perceived advantages and limitations.

**Results:**

A total of 142 specialists (16%–20% estimated response rate) responded (83 male, 59 female). Most were aged 30–39 years (47.2%), had 11–20 years of experience (40.8%), and worked in private practice (59.9%). CAT was used by 69% of respondents; 35.9% treated <10 new CAT cases annually, while 7.7% treated >50. For 51.4%, CAT comprised about 10% of their caseload; only 2.8% reported CAT use in >50% of cases. CAT was used mainly in adults and least in mixed dentition/adolescents (*P* < 0.001). Preferred indications were mild crowding (<4 mm; 81.6%), spacing/diastema (63.1%), anterior crossbite (44.3%), and mild open bite (1–2 mm; 38.6%). CAT was favoured for single-extraction or space-opening cases rather than complex extractions or orthognathic surgery. About 8.5% reported using a hybrid approach, consisting of CAT in one arch combined with fixed appliance therapy in the opposing arch. Elastics were the most common adjunct. Intraoral 3D scanners were the most common method (68.1%) for taking records among respndents (*n* = 141). Approximately, 49.3% and 19% of aligners were provided by local manufacturers or produced in-office, respectively. Most orthodontists recognised biomechanical limitations of CAT (agree and strongly agree), with 73.3% reporting limited control of tooth movement, 77.5% inadequate torque expression, 88.7% difficulty achieving certain tooth movements, and 88.8% a limited scope for complex cases. Furthermore, 68.1% believed CAT may require longer treatment times than fixed appliances, and 83.1% agreed that CAT often necessitates greater interproximal reduction than fixed appliance therapy.

**Conclusion:**

CAT was mainly used for mild malocclusions, supported by expanding local aligner manufacturers and in-office aligner production.

## Background

Clear aligner therapy (CAT) has become integral in modern orthodontics, providing clinicians with an alternative tool for managing tooth movement and enhancing patient comfort and aesthetics (1–3). Global adoption of these technologies, however, is shaped not only by clinical considerations but also by broader economic and political factors.

There are some studies reporting the current practice of CAT in the UK, Ireland, Australia, Canada, and New Zealand (2–6); however, there is no published evidence from Iran on how specialist orthodontists adopt these modalities across indications, training routes, brands/systems, and protocols. Studying specialist orthodontists in Iran offers an important opportunity to document how orthodontists chose and incorporated CAT in their orthodontic treatment protocols and sourcing from domestic/international manufacturers.

The study objectives were (1) to quantify the frequency, protocols, and evaluate perceptions of the advantages and disadvantages of CAT, and the experienced complications and risks associated with CAT among specialist orthodontists in Iran (2), to assesses the clinical indications for CAT among Iranian orthodontists when treating different malocclusions, and to determine the extent to which Iranian homegrown companies (e.g., domestic aligner systems), if any, substitute for international providers and exploring the variation in protocols, training, and device/systems used in Iran and (3) determine the frequency of CAT use in different age groups (mixed dentition, adolescents/teenagers, and adults) and identifies the most common adjunctive devices [Temporary Anchorage Devices (TADs), Elastics] and appliances (fixed braces and functional appliances) used with CAT.

### Methodology

Ethical approval for this study was granted by the Cardiff University Dental School Research Ethics Committee (ref: 2519).

### Study design

This study forms part of the *Cardiff University International Aligner Survey: Orthodontists’’ Practices and Perceptions*. A web-based cross-sectional questionnaire comprising 24 items selected from previously validated studies in USA (2) and UK (3) was administered to specialist orthodontists practising in Iran. The survey was conducted in English; participation was voluntary and anonymous.

### Pilot testing and cultural adaptation

A pilot phase (*n* = 10) was conducted in Iran prior to full deployment to assess completion time, item clarity, and instrument refinement. Face validity and content clarity were assessed during pilot testing and participants reported comfort with the English-language format, confirming its suitability for the target population. Each survey was estimated to take approximately 10–15 min to complete.

### Setting and population

#### Setting

Specialist orthodontic practices, university or teaching hospitals, and public orthodontic clinics in Iran.

#### Population

The study population comprised licensed specialist orthodontists practising in Iran.

#### Inclusion criteria

Board-certified or registered orthodontic specialists currently engaged in active clinical practice in Iran.

#### Exclusion criteria

Orthodontic residents or trainees not yet holding specialist status, and retired practitioners no longer treating patients.

### Sampling frame and recruitment

Participants were recruited through a mixed-mode electronic strategy (7), combining direct invitations via professional social media platforms with email invitations distributed to publicly available addresses from universities and professional networks. This approach was selected to maximise coverage of the target professional population and reduce non-response bias associated with single-mode recruitment. As a comprehensive national register of specialist orthodontists in Iran is not publicly available and the medical council register shows the number of all registered Iranian orthodontists that may include deceased or migrated orthodontists. This convenience sampling approach was used to reach as broad a segment of the eligible population as possible.

### Survey instrument and measures

A structured questionnaire with a landing page emphasising the voluntary and anonymous nature of participation collected the following variables:

#### Demographics and practice characteristics

Age group, gender, years in orthodontic practice, practice setting (private, university/public, or mixed), and practice location (city).

#### Training and supply environment

Training providers, procurement channels (major international brands, domestic manufacturers, in-office fabrication).

#### CAT utilisation

Annual case starts; proportion of overall practice; proportion of CAT use across age groups [mixed dentition (7–10 years), adolescents (11–18 years), and adults (>18 years)].

#### Case selection

Malocclusion types including crowding/spacing, deep/open bite, expansion, crossbites, extraction cases, space opening, and combined orthodontic-surgical cases.

#### Adjuncts

Use of TADs, elastics, fixed appliances (metal/ceramic), and functional appliances.

#### Systems and data acquisition

CAT provider systems (e.g., Invisalign, Spark, ClearCorrect, domestic Iranian systems, in-office aligners); digital scanning vs. impressions; review intervals and retention protocols; training routes.

#### Perceptions

Perceived advantages and disadvantages, including aesthetics, comfort, oral hygiene, treatment outcomes, limitations (torque control, complexity), duration, IPR, risks (pain, infection, emergencies, compliance, aligner loss, patient complaints).

### Data management and anonymisation

No identifiable personal data were collected (e.g., names, registration numbers, or clinician-specific details). Practice location was recorded at city level only to minimise identification risk. All responses were anonymised at the point of collection. Data are stored securely on password-protected devices with encrypted backup. Access to raw data is restricted to the research team.

### Statistical analysis

All statistical analyses were conducted using SPSS (version 26, IBM, Armonk, NY). Descriptive statistics were used to calculate frequencies and percentages. Chi-square tests were carried out to determine whether differences existed in the provision of clear aligner therapy (CAT) between male and female orthodontists with respect to respondents’ age, years in practice, practice setting, and number of CAT cases provided per year. Chi-square tests were also performed to assess whether differences existed in the percentages of patients treated with CAT across the following age groups: children in the mixed dentition (7–10 years), adolescents (11–18 years), and adults (>18 years).

## Results

### Basic demographics of respondents

Data were collected between October 2025 and January 2026. The sample comprised 142 specialist orthodontists (83 males and 59 females). Based on the total number of invitations distributed across recruitment channels (451 invitations through the Iranian Association of Orthodontists membership list and 424 invitations distributed through social media and institutional email networks), a maximum of 875 invitations were issued. Assuming no overlap between these recruitment channels, the crude response rate was approximately 16.2%:

142/875×100=16.2%

However, overlap between recruitment sources was considered likely because many practising orthodontists may have been contacted through both the association membership list and social media/email distribution routes. Consequently, the true number of unique invitees was probably lower than 875, resulting in a somewhat higher actual response rate. Assuming a modest overlap of approximately 10%–20% between recruitment sources, the estimated number of unique invitees would range from approximately 700 to 800 orthodontists, yielding a higher estimated response rate of approximately 18%–20%:

142/800×100=17.8%

142/700×100=20.3%

National registries list approximately 961 orthodontists in Iran; however, these registries are not regularly updated and likely include individuals who are deceased, retired, emigrated, or no longer clinically active. Using the registry figure as the broadest available denominator, the study sample represented approximately 14.8% of all registered orthodontists in Iran:

142/961×100=14.8%

As the true number of actively practising orthodontists is likely lower than 961, the proportion of the active orthodontic workforce represented by the survey was probably higher. For example, if the active workforce comprised approximately 800–850 orthodontists, the survey sample would represent approximately 16.7–17.8% of practising orthodontists in Iran:

142/850×100=16.7%

142/800×100=17.8%

Therefore, the overall survey response rate is estimated to be approximately 16%–20%. Not all respondents responded to every question and the percentages given related to the proportion of respondents who answered the relevant questions.

Most respondents were aged 30–39 years (47.2%), had 11–20 years of orthodontic practice experience (40.8%), and worked in private practice (59.9%) (Table 1). There were no significant gender differences in age, years of orthodontic practice experience, type of orthodontic practice, or number of CAT starts per year (Table 1; *P* > 0.05). Most respondents were from Tehran (31.7%), followed by Isfahan (7.0%), Mashhad (7.0%), Shiraz (4.2%), Ahvaz (4.2%), and Rasht (3.5%). The remaining respondents were from other cities, each represented by one to four participants.

**Table 1:** Age and gender distribution and orthodontic practice experience of respondents, N (%).

Age*	Male	Female	Total
<30	4 (4.8)	4 (6.8)	8 (5.6)
30–39	32 (38.6)	35 (59.3)	67 (47.2)
40–49	31 (37.3)	13 (22.0)	44 (31.0)
50–59	9 (10.8)	5 (8.5)	14 (9.9)
≥60	7 (8.4)	2 (3.4)	9 (6.3)
Years in Practice**			
<5	9 (10.8)	11 (18.6)	20 (14.1)
5–10	21 (25.3)	22 (37.3)	43 (30.3)
11–20	37 (44.6)	21 (35.6)	58 (40.8)
>20	16 (19.3%)	5 (8.5)	21 (14.8)
Practice setting***			
Private Practice	50 (60.2)	35 (59.3)	85 (59.9)
University/Public	3 (3.6)	2 (3.4)	5 (3.5)
Mixed	30 (36.1)	22 (37.3)	52 (36.6)
How many CAT cases do you start per year?****			
I do not currently use CAT for orthodontic treatment	27 (32.5)	17 (28.8)	44 (31.0)
<10	27 (32.5)	24 (40.7)	51 (35.9)
10–25	11 (13.3)	8 (13.6)	19 (13.4)
26–50	9 (10.8)	8 (13.6)	17 (12.0)
>50	9 (10.8)	2 (3.4)	11 (7.7)
Total	83 (58.5)	59 (41.5)	142 (100)

* Pearson Chi-Square=7.744, *P* = 0.108 (Likelihood ratio significance).

** Pearson Chi-Square=6.529^a^, *P* = 0.089.

*** Pearson Chi-Square=0.022^a^, *P* = 0.989 (Likelihood ratio significance).

**** Pearson Chi-Square=3.479, *P* = 0.481.

### CAT usage and training among respondents

CAT utilisation varied substantially among respondents, ranging from non-users (31%) to high-volume providers treating more than 50 new CAT cases annually (7.7%) (Table 1).

Respondents received CAT training through different means such as aligner manufacturer's course/workshop (50%), Online training/webinar (37.3%), and Postgraduate orthodontic training (37.3%).

For 51.4% of respondents, CAT patients accounted for approximately up to 10% of their practice caseload, whereas only 2.8% reported that CAT comprised 50% or more of their total caseload (Table 2).

**Table 2:** Method of receiving training for CAT among Iranian orthodontists, distribution of practice caseload treated with CAT, and types of CAT combinations provided.

Method of receiving training for CAT*	N (%)
Undergraduate dental training	4 (2.8)
Postgraduate orthodontic training (residency)	28 (19.7)
Manufacturer's course/workshop	71 (50)
Online training/webinar	53 (37.3)
Other (e.g., learned from a colleague)	44 (31)
I do not currently use CAT for orthodontic treatment	32 (22.5)
Overall practice percentages of clear aligner patients	
I do not currently use CAT for orthodontic treatment	45 (31.7)
<10%	73 (51.4)
10%–25%	15 (10.6)
26%–50%	5 (3.5)
>50%	4 (2.8)
Total	142
Type of CAT you have provided so far	
Upper and lower CAT	81 (57.0)
Mostly upper CAT	6 (4.2)
Mostly lower CAT	2 (1.4)
Upper or lower CAT combined with fixed appliance therapy	12 (8.5)
I do not currently use CAT for orthodontic treatment	41 (28.9)
Total	142

* Multiple answers allowed, not all respondents answered this question (*n* = 141).

Overall, 37.3% believed that CAT provision had increased their orthodontic caseload, and 73.9% anticipated that their CAT provision would increase over the next five years. The most common form of CAT provision was combined upper and lower CAT; however, 8.5% reported using a hybrid approach, consisting of CAT in one arch combined with fixed appliance therapy in the opposing arch (Table 2).

According to the survey data, the percentage of patients treated with CAT differed significantly among children in the mixed dentition (7–10 years), adolescents (11–18 years), and adults (>18 years) (*P* < 0.001). CAT was most frequently used in adults and least commonly used in the mixed dentition group (Table 3).

**Table 3:** Percentage [n (%)] of patients treated with CAT in children in the mixed dentition (7–10 years), adolescents (11–18 years), and adults (>18 years).

Percentage	Mixed dentition (7–10 years) a	Adolescents (11–18 years) b	Adults (>18 years) c
< 10%	64 (45.1)	70 (49.3)	48 (33.8)
10–25%	1 (0.7)	17 (12.0)	27 (19.0)
26–50%	1 (0.7)	3 (2.1)	11 (7.7)
> 50%	1 (0.7)	2 (1.4)	16 (11.3)
I do not currently use CAT for orthodontic treatment	74 (52.1)	50 (35.2)	40 (28.2)
Missing	1		
	142	142	142

a vs. b, Pearson Chi-Square=108.381, *P* < 0.001.

a vs. c, Pearson Chi-Square=82.758, *P* < 0.001 (Likelihood ratio significance).

b vs. c, Pearson Chi-Square=169.740, *P* < 0.001 (Likelihood ratio significance).

### Orthodontist preferences for CAT or FAT in treating certain occlusal problems

**Figure 1:**
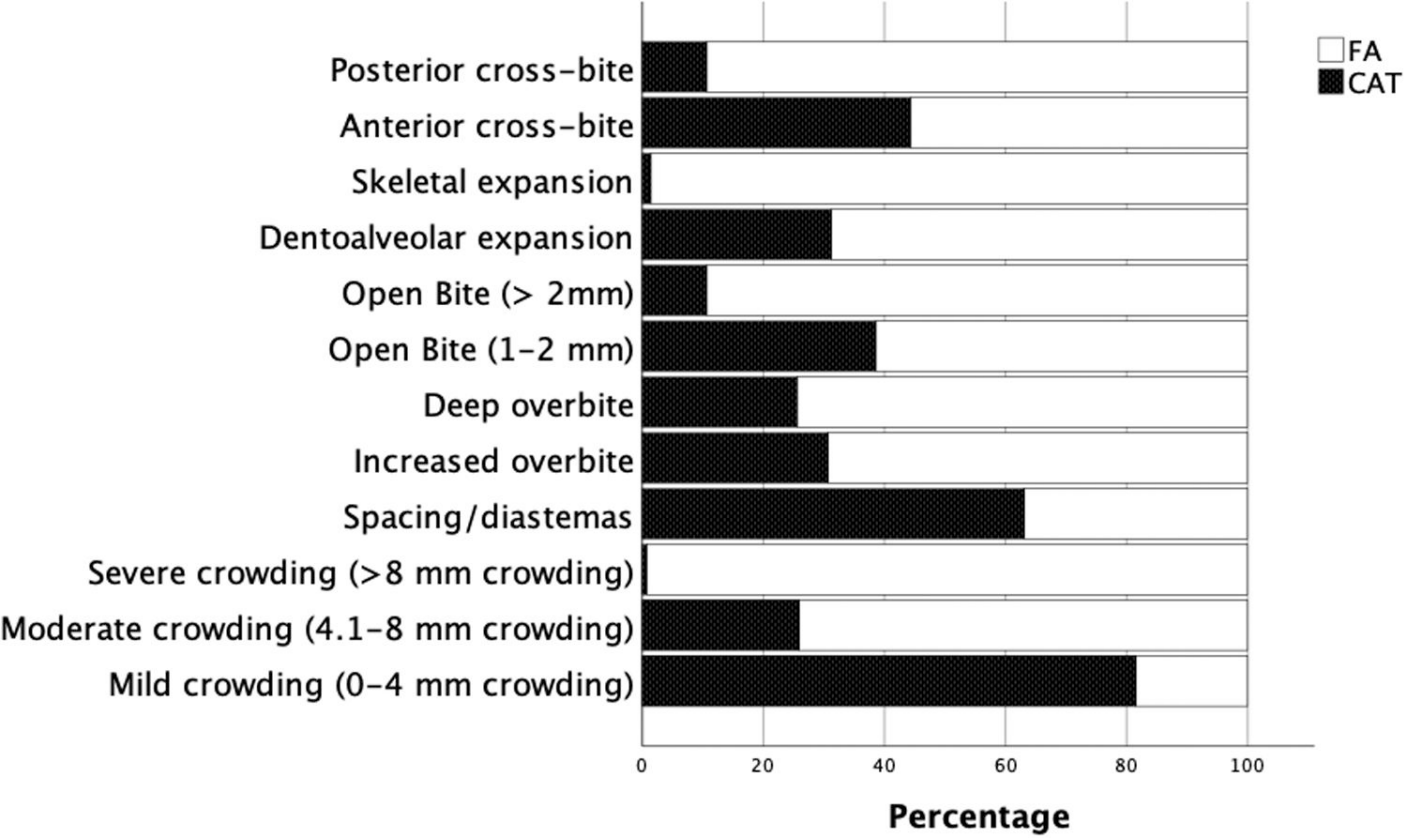
Preferences (%) for CAT or FAT among respondents when treating specific occlusal problems.

As shown in Figure 1, 81.6% of Iranian orthodontists preferred to use CAT for treating mild crowding (< 4 mm), followed by spacing or diastema (63.1%), anterior crossbite (44.3%), mild open bite (1–2 mm) (38.6%), dentoalveolar expansion (31.2%), increased overbite (30.7%), moderate crowding (4.1–8 mm) (25.9%), deep overbite (25.5%), open bite > 2 mm (10.6%), posterior crossbite (10.6%), skeletal expansion (1.4%), and severe crowding > 8.1 mm (0.7%). Table 4 shows the treatment scenarios and adjuncts used with CAT. Iranian orthodontists were more inclined to use CAT for single-extraction or space-opening cases than for multiple extractions (e.g., upper and lower premolars), molar extractions, or orthognathic surgery cases.

**Table 4:** Distribution (%) of treatment scenarios and adjuncts used with CAT.

Treatment scenarios treated with CAT	Always	Mostly	Sometimes	Rarely	Never
Single-tooth extraction cases (e.g., one lower incisor)	2.3%	6.9%	30.8%	23.1%	36.9%
Multiple extractions (e.g., 2 upper + 2 lower premolars)	0.0%	0.0%	3.8%	18.5%	77.7%
Molar extractions (e.g., poor prognosis 1st molars or open bite closure)	0.0%	0.0%	1.5%	14.6%	83.8%
Space opening (e.g., for missing teeth/implants)	1.5%	2.3%	30.0%	22.3%	43.8%
Combined ortho-surgery cases	0.0%	0.8%	5.4%	14.7%	79.1%

Adjunctive used with CAT	Always	Mostly	Sometimes	Rarely	Never
Temporary Anchorage Devices (TADs)	0.8%	1.6%	39.8%	23.4%	34.4%
Elastics	8.7%	18.1%	41.7%	6.3%	25.2%
Fixed appliances (metal braces)	0.8%	3.9%	39.8%	27.3%	28.1%
Fixed appliances (ceramic braces)	0.8%	2.3%	19.5%	22.7%	54.7%
Functional appliances	0.0%	2.3%	14.8%	18.8%	64.1%

Among the orthodontists surveyed, elastics were the most used adjunct with CAT, while temporary anchorage devices (TADs) are used selectively (mostly or sometimes by about 40%), and fixed appliances (especially ceramic) and functional appliances are rarely or never combined with CAT by the large majority. Approximately 49.3% and 19% of the clear aligners were provided by local aligner manufacturers or produced in-office, respectively (Table 5).

**Table 5:** Distribution [n (%)] of aligner systems used, methods of obtaining records for CAT, retainer types provided after CAT, and CAT review intervals. Not all respondents answered all questions.

Aligner system used*	n (%)
Invisalign	10 (7.6)
Spark	0
ClearCorrect	1 (0.8%)
Other international aligner providers	7 (4.9%)
Local aligner manufacturers	70 (49.3%)
In-office aligners (you make them in the office)	27 (19%)
I do not currently use CAT for orthodontic treatment	39 (28.9%)
Method of obtaining records for CAT planning	
Intraoral 3D scanner	96 (68.1)
Impressions with Polyvinyl Siloxane or other materials	4 (2.8)
Both	5 (3.5)
I do not currently use CAT for orthodontic treatment	36 (25.5)
Retainer type provided after CAT*	
Clear (vacuum-formed) retainers	76 (53.9)
Hawley type retainers	7 (5)
Fixed bonded retainers	75 (53.2)
I do not currently use CAT for orthodontic treatment	38 (27)
Review interval for patients during CAT treatment	
Every 4–6 weeks	56 (43.4)
Every 8–10 weeks	31 (24)
Every 12 + weeks	4 (3.1)
Only if there is a problem—otherwise, I give the patient all the aligners and see them at the end of treatment	1 (0.8)
I do not currently use CAT for orthodontic treatment	37 (28.7)

* Multiple answers allowed.

The Iranian orthodontists also reported using Invisalign (7.6%), ClearCorrect (0.8%), and other international aligner providers (4.9%) (Table 5). Intraoral 3D scanners were the most common method (68.1%, *n* = 141) for taking records for CAT (Table 5).

Clear (vacuum-formed) retainers (53.9%) and fixed bonded retainers (53.2%) were the most common retainer types provided after CAT (Table 5).

Review intervals for patients during CAT were mainly every 4–6 weeks (43.4%) or every 8–10 weeks (24%) (Table 5).

### Orthodontist perception of advantages and disadvantages

Orthodontists generally perceived CAT as advantageous in terms of aesthetics, oral hygiene maintenance, patient comfort, and reduced emergency visits, with strong agreement particularly for improved aesthetics and hygiene (Table 6). Most orthodontists recognised biomechanical limitations of CAT (agree and strongly agree), with 73.3% reporting limited control of tooth movement, 77.5% inadequate torque expression, 88.7% difficulty achieving certain tooth movements, and 88.8% a limited scope for complex cases. Furthermore, 68.1% believed CAT may require longer treatment times than fixed appliances, and 83.1% agreed that CAT often necessitates greater interproximal reduction than fixed appliance therapy. CAT was also widely perceived as more costly, more time-consuming in some cases, and requiring high patient compliance, with a notable risk of aligner loss or damage. In contrast, perceptions regarding higher relapse rates, increased patient complaints, and potential microplastic release were largely neutral or uncertain.

**Table 6:** Perception (%) of orthodontist regarding advantages and disadvantages of CAT. Not all respondents answered all questions.

Perceived advantages and disadvantages of CAT	Strongly agree	Agree	Neither agree nor disagree	Disagree	Strongly disagree
CAT offers better aesthetics compared to traditional braces.	38.0%	43.4%	9.3%	5.4%	3.9%
CAT provides greater patient comfort during treatment.	10.8%	55.4%	20.8%	10.0%	3.1%
CAT allows for easier oral hygiene maintenance.	41.5%	50.0%	6.2%	0.8%	1.5%
CAT results in fewer emergency visits compared to traditional braces.	18.5%	53.1%	21.5%	6.2%	0.8%
Patient acceptance of CAT treatment is higher than for other orthodontic options.	6.1%	31.3%	43.5%	16.0%	3.1%
CAT can be effectively used for children who dislike traditional fixed braces.	1.5%	21.5%	41.5%	30.0%	5.4%
CAT provides superior treatment outcomes in appropriate cases.	2.3%	19.1%	26.0%	26.0%	26.7%
CAT provides limited control over tooth movement.	28.2%	45.1%	18.3%	8.5%	0.0%
CAT has inadequate torque expression compared to fixed braces.	31.7%	45.8%	10.6%	11.3%	0.7%
CAT has a limited scope for treating complex orthodontic cases.	43.7%	45.1%	6.3%	4.9%	0.0%
CAT treatment can take longer for some cases compared to fixed appliances.	22.0%	46.1%	20.6%	9.9%	1.4%
Certain tooth movements are difficult to achieve with CAT.	41.5%	47.2%	9.2%	2.1%	0.0%
The relapse rate after CAT treatment is higher than with other methods.	2.8%	12.8%	46.1%	32.6%	5.7%
CAT often requires more interproximal reduction (IPR) than fixed appliance therapy.	23.9%	59.2%	9.9%	6.3%	0.7%
The cost of CAT is higher than that of traditional braces.	50.7%	41.5%	4.2%	3.5%	0.0%
Successful CAT treatment requires high patient compliance.	69.0%	26.8%	3.5%	0.7%	0.0%
There is a risk of aligners being lost or damaged during treatment.	26.8%	51.4%	14.8%	7.0%	0.0%
Patients tend to have more complaints during or after CAT treatment than with fixed appliances.	8.5%	17.0%	38.3%	32.6%	3.5%
CAT may cause microplastic release in saliva, potentially affecting general health.	2.1%	16.2%	55.6%	20.4%	5.6%

## Discussion

Clear aligner therapy (CAT) has become a popular alternative to fixed braces, with previous studies indicating that 65%–95% of orthodontists in several countries incorporated it into their practice, according to multiple recent surveys (2, 3, 5, 6, 8, 9).

To the authors knowledge, this was the first comprehensive survey to investigate CAT practices and protocols among Iranian specialist orthodontists. The present study evaluated orthodontists’ usage patterns, preferences, and perceptions regarding CAT rather than objective clinical effectiveness or treatment outcomes. Our data suggested that CAT was used by most respondents (69%) in Iran. This figure is comparable to the recent study in Turkey (69.5%) (10), lower than that reported for the United Kingdom (77.3%) (3) and another study from Turkey (79.5%) (11), and substantially lower than the 92%–93% recorded in Australia (2) and the United States (12).

Usage patterns varied considerably: about 35.9% of respondents reported starting fewer than 10 new CAT cases per year, while 7.7% reported more than 50 starts annually. Almost half (49.3%) reported treating 1–25 CAT cases per year. In comparison, for the UK and Ireland, almost half (48.1%) reported treating 1–20 CAT cases annually (3), and 72% treated 1–50 cases per year; the corresponding figure for Iran was 61.3%, which is lower. The recent survey of North American orthodontist suggested that about 25% of all practice caseloads were treated with CAT, and it is mostly patients with mild-to-moderate crowding, spacing, and anterior open bite (13, 14). There is a trend for younger orthodontists to fabricate in-office aligners and plan to do so more frequently in the future (13, 14).

The relatively low response rate of approximately 16%, similar to a Turkish (4.1%) study (10), is another potential limitation. As noted by Meade et al. (3), this rate is comparable to surveys involving British Orthodontic Society members (15%–20.1%) and surveys conducted in the UK, Canada, USA, and France concerning orthodontic practice protocols (1.6%–18.1%) (3, 9, 10, 12, 15–18).

Similar to findings in the UK and Ireland, among respondents using CAT, the patient cohort most frequently treated comprised adults (3). Due to embargoes, the Invisalign system was not widely used in Iran, with only 7% of respondents reporting its use. In contrast, about 81.25% in the USA (9), over 80% in the UK and Ireland survey (3) and 63% in an Australian survey (2) reported using Invisalign. Interestingly, local aligner manufacturers and in-office aligner production were predominant, accounting for approximately 49.3% and 19% of cases, respectively.

Most respondents reported being uncomfortable treating cases with severe crowding. Additionally, 77.7% and 83.8% indicated that they never performed CAT in cases involving multiple extractions or molar extractions, respectively. Similarly, the UK and Ireland study (3) found that over 60% of respondents rarely or never extracted premolar teeth in association with CAT.

Consistent with the UK and Ireland findings (3), most respondents perceived that CAT did not provide superior treatment outcomes compared with fixed appliance therapy (FAT). These findings reflect clinician perceptions rather than objective clinical outcome assessment. Previous studies have reported challenges in achieving certain orthodontic movements and managing complex malocclusions with CAT (19–26).

The UK and Ireland study (3) reported that about 25% of respondents incorporated both CAT and FAT in their initial digital treatment plans. A higher proportion of hybrid approaches (50%–60%) was reported in Australia and the United States (2, 12). When we looked at clinical preferences, 44.5% of respondents preferred combining fixed metal braces with CAT (ranging from sometimes to always), 22.6% combining ceramic fixed braces with CAT, and 17.1% combination of functional appliances with CAT, though to a lesser extent. In addition, about 8.5% of Iranian orthodontists reported using a hybrid approach, consisting of CAT in one arch combined with fixed appliance therapy in the opposing arch. Kravits et al. (27) highlights that hybrid CAT and fixed braces use occurs in about 17.2% of Invisalign-initiated cases as a rescue or finishing strategy, underscoring limitations of aligners alone in some patients and the resulting need for combined modalities to achieve optimal outcomes. Recent systematic reviews have similarly reported limitations in predictability for certain tooth movements, including torque control, extrusion, and complex bodily movements with CAT (28–31).

Our survey revealed that for 51.4% of respondents, less than 10% of their practice caseload consisted of patients treated with CAT; however, only 2.8% reported caseloads where more than 50% of cases involved CAT.

CAT was most preferred for relatively simple malocclusions, particularly mild crowding and spacing. Approximately 81.6% of Iranian orthodontists preferred to use CAT for treating mild crowding (< 4 mm), followed by spacing or diastema (63.1%), anterior crossbite (44.3%), and mild open bite (1–2 mm) (38.6%). However, preference for using CAT in other malocclusion types was relatively low, including dentoalveolar expansion (31.2%), increased overbite (30.7%), moderate crowding (4.1–8 mm) (25.9%), deep overbite (25.5%), open bite > 2 mm (10.6%), posterior crossbite (10.6%), skeletal expansion (1.4%), and severe crowding > 8.1 mm (0.7%). Although the CAT study conducted in the USA did not use the same malocclusion classification as the present study, it reported a higher preference for treating a wider range of malocclusion types, including more complex cases, using CAT (9).

A survey of USA orthodontists reported only 4.3% used CAT for orthognathic cases (9), similarly very few of the Iranian orthodontists (6.2%, ranging from sometimes to always) used CAT for orthognathic cases. The present findings suggest that CAT was not used in 52.1%, 35.2%, and 28.2% of children with mixed dentition, adolescents, and adults, respectively. A similar trend has previously been reported among orthodontists in the USA (1, 9). Another study of paediatric dentists in India revealed that 63.9% had never used clear aligners in mixed dentition (32), although the use of CAT in mixed dentition may differ among Indian orthodontists.

Provision of clear (vacuum-formed) and fixed bonded retainers was equally popular following CAT; however, a study conducted in the USA reported that 83.12% and 43.13% of respondents preferred vacuum-formed and fixed bonded retainers, respectively (9).

### Limitations

This study has several limitations that should be considered when interpreting the findings. First, selection bias may have occurred because participation was voluntary, and orthodontists with a particular interest in the survey topic may have been more likely to respond. Although multiple recruitment channels were used to enhance coverage, the exact number of actively practising orthodontists in Iran remains unknown, limiting the precision of the estimated response rate. Consequently, the representativeness and generalisability of the findings to all orthodontists practising in Iran may be limited. Second, the survey was conducted in English, which may have introduced language bias by excluding or discouraging participation from orthodontists with limited English proficiency, potentially affecting the representativeness of the sample. Third, dissemination via social media and electronic platforms may have preferentially recruited younger or more digitally engaged orthodontists, introducing potential selection bias.

However, responses were received from orthodontists working across a wide range of geographical locations, which similar studies have rarely reported, and clinical environments such as private practice, university and mixed practice, suggesting that the findings are likely to have external validity. In addition, formal psychometric validation of the questionnaire, including reliability testing and internal consistency assessment, was not undertaken and should be considered in future studies. Given the exploratory nature of the study and moderate sample size, multivariable modelling was not performed; therefore, the findings should be interpreted as descriptive associations rather than independent predictors. Effect size measures were not calculated and should be considered in future studies.

With a larger sample size a simple logistic retrogression model could be planed, with CAT user vs. non-user as the outcome, investigating predictors such as age, years of clinical experience, private practice, and raining type.

Similar to other surveys response quality may have been affected by satisficing (33–36), where respondents forego the full cognitive effort required for optimal survey responding, including careful question interpretation, memory retrieval, and judgement formation, in favour of a more cursory approach. For the present study, satisficing may partly explain inconsistencies observed between conceptually related items, such as those pertaining to annual CAT frequency and the proportion of patients receiving CAT, where a small number of respondents appeared to provide contradictory answers across questions. Future studies could employ bilingual surveys and using updated professional registries to improve participation and reduce potential sources of bias.

Despite the limitations, the inclusion of respondents from diverse practice settings and professional backgrounds supports the generalisability of the results to orthodontists currently practising in Iran, although caution is warranted. Future studies could benefit from administering bilingual surveys and using updated professional registries to improve participation and reduce potential sources of bias.

## Conclusion

The majority of survey respondents in Iran reported providing CAT, with a wide variation in treatment numbers and protocols. Overall, approximately one-third of respondents did not use CAT as part of their orthodontic practice. Among those who did, the most frequently prescribed systems were local aligner manufacturers and in-office aligner production, accounting for approximately 49.3% and 19% of cases, respectively. Most respondents perceived that CAT did not produce superior outcomes compared with fixed appliance therapy.

## Data Availability

The raw data supporting the conclusions of this article will be made available by the authors, without undue reservation.
